# A Case of Pseudohypoparathyroidism With Unusual Presentation and Novel Genetic Mutation

**DOI:** 10.7759/cureus.111078

**Published:** 2026-06-18

**Authors:** Reem Al-Amri, Ziad A Taher, Rawan A Alnajashi, Atheer A Alqurashi, Hend Al-Zanbaqi

**Affiliations:** 1 Endocrinology, National Guard Health Affairs, Jeddah, SAU; 2 Department of Medicine, King Abdullah International Medical Research Center, Jeddah, SAU; 3 Department of Medicine, National Guard Health Affairs, Jeddah, SAU; 4 Internal Medicine, National Guard Health Affairs, Jeddah, SAU; 5 Endocrinology, Diabetes and Metabolism, National Guard Health Affairs, Jeddah, SAU

**Keywords:** albright hereditary osteodystrophy, fine needle aspiration, parathyroid hormone, pseudo-pseudohypoparathyroidism, thyroid-stimulating hormone

## Abstract

Pseudohypoparathyroidism is a really rare inherited disorder characterized by either unresponsiveness or targeted organ resistance to the parathyroid hormone, classified either biochemically or by phenotype characteristics. Here, we report an atypical presentation of Albright hereditary osteodystrophy. An underweight 18-year-old male presented with painful subcutaneous nodules that progressively appeared over a period of 10 years and were confirmed by fine needle aspiration, and the pathology report indicated osteoma cutis.

## Introduction

Pseudohypoparathyroidism (PHP) is a very rare inherited disorder demonstrated by unresponsiveness or by target organ resistance to parathyroid hormone (PTH), leading to abnormalities in calcium and phosphate regulation. Biochemically, patients usually present with high PTH levels with hypocalcemia and hyperphosphatemia. Some types of PHP are linked to Albright hereditary osteodystrophy (AHO), a group of physical characteristics associated with changes in the GNAS gene [1]. The syndrome mimics hypoparathyroidism, with patients experiencing hypocalcemia and hyperphosphatemia. However, patients exhibit elevated instead of low serum PTH levels. PHP is typically classified as type 1 or type 2, with type 1 further categorized as 1a, 1b, or 1c. The cyclic adenosine monophosphate (cAMP) response to G protein activation is normal in type 2 and abnormal in type 1, thereby distinguishing the two types. Those with PHP 1a and 1c can exhibit multi-hormone resistance, whereas 1b is localized only to the kidney [1].

Patients with type 1a and 1c are also categorized by the variable expression of a set of physical features, referred to as Albright hereditary osteodystrophy (AHO), which includes the premature closure of growth plates, short bones, short stature, a stocky build, ectopic ossifications, and other poorly defined abnormalities. In some patients, the physical features of AHO might be present in the absence of hormone resistance [2]. Moreover, based on the extent of ectopic ossifications and number of AHO features, patients might be classified as having pseudo-pseudo hypoparathyroidism (PPHP; Online Mendelian Inheritance in Man (OMIM) #612463), progressive osseous heteroplasia (POH; OMIM #166350), or osteoma cutis, depending on the number of AHO features present and the extent of ectopic ossifications [2].

## Case presentation

**Figure 1:**
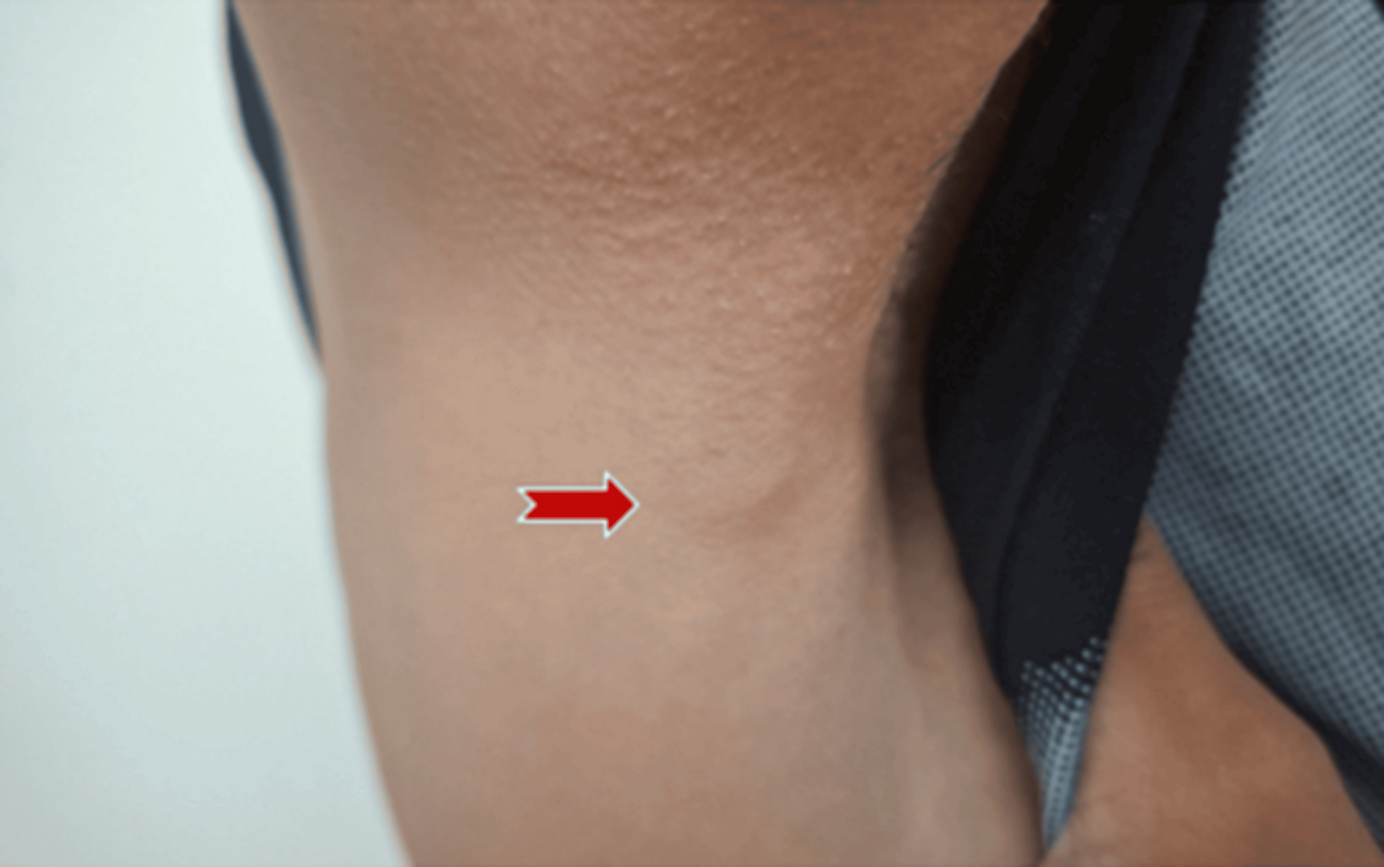
Subcutaneous nodule in right axilla appearing initially

**Figure 2:**
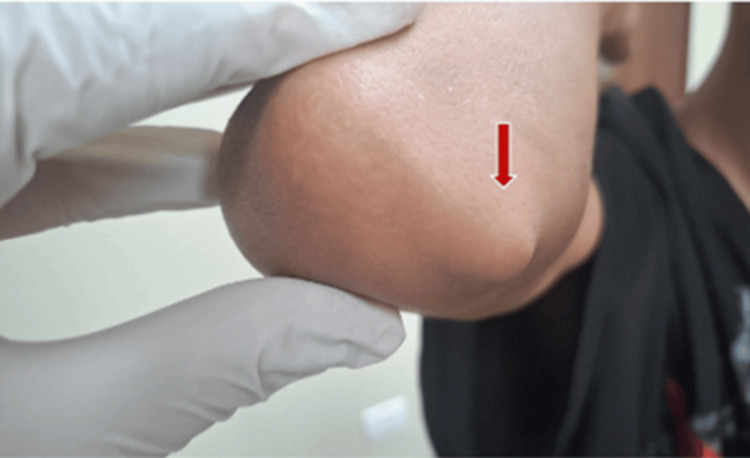
Subcutaneous nodule in extensor surface of right elbow

**Figure 3:**
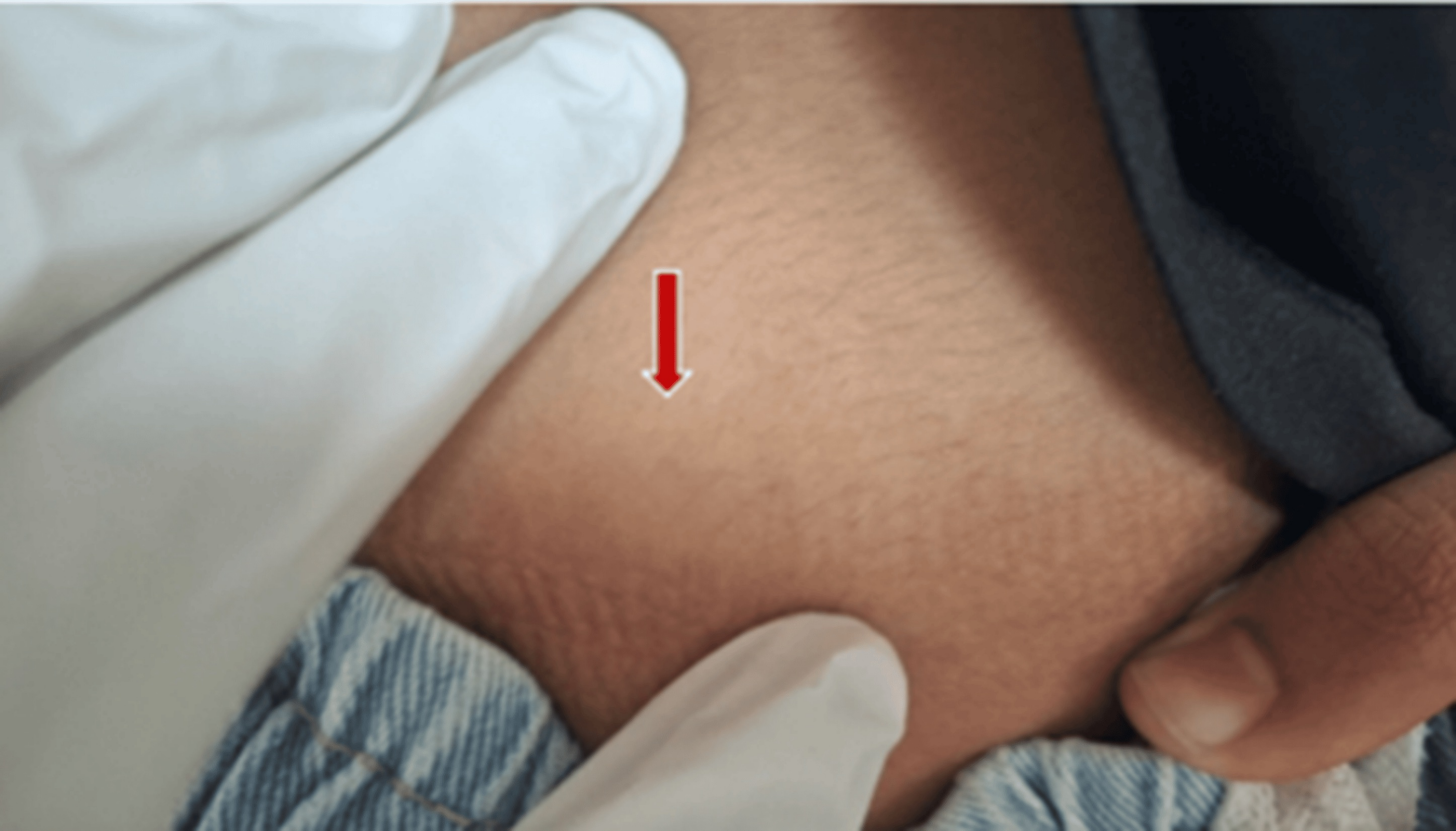
subcutaneous nodule in right lower quadrant abdomen

An 18-year-old male, the eldest of five siblings from consanguineous parents, presented initially with painful skin colored subcutaneous nodules that started to appear 10 years prior. Started as small nodules, not painful unless he puts pressure on them, which over the past 10 years have increased in size, becoming painful. Nodules appeared for the first time in his right axilla (Figure 1), then on the extensor surface of his right elbow three years later (Figure 2), followed by the right lower quadrant abdomen (Figure 3) and, finally, the posterior surface of his right leg two years ago. The nodules grew slowly and were painless, except for the one in his abdomen, which became painful, especially when he wore his pants around it, prompting him to seek medical advice. 

The patient denied any history of peripheral or oral numbness, muscle spasm, or seizures. He had no known medical illnesses or prior surgeries. Family history revealed that one of his four siblings had similar skin lesions (two nodules, one on her scalp and another on her foot), raising the possibility of familial inheritance. His father and mother are consanguineous.

Diagnostic assessment

Upon examination, the patient was found to be underweight (36 kg; BMI 13 kg/m^2^) and to be 169 cm tall. His nodules were hard, firm, immobile, painless, and smooth-margined, except for the one in his abdomen, which was painful to pressure. He was negative for Trousseau’s and Chvostek’s signs. The laboratory examination yielded the following results (Table 1).

**Table 1:** Laboratory examination results SI: International System of Units.

Laboratory Test	Patient Value	Reference Range	Interpretation
Parathyroid Hormone (PTH)	104.90 pg/mL (SI: 11.1pmol/L)	16.04-60.38 pg/mL (SI: 1.7-6.4 pmol/L)	Elevated Parathyroid Hormone (PTH)
Adjusted Calcium (Adj Ca)	8.7 mg/dL (SI: 2.18 mmol/L)	8.6-10.2 mg/dL (SI: 2.15-2.55 mmol/L)	Low-normal calcium level
Phosphorus	5.3 mg/dL (SI: 1.7 mmol/L)	2.5-4.5 mg/dL (SI: 0.8-1.44 mmol/L)	Elevated phosphorus level (hyperphosphatemia)
25-Hydroxyvitamin D	85.3 nmol/L (34.1 ng/mL)	50-125 nmol/L (20-50 ng/mL)	Sufficient vitamin D status
1,25-Dihydroxyvitamin D	118 pmol/L (49 pg/mL)	48-190 pmol/L (20-79 pg/mL)	Within normal range
Growth hormone (GH)	1.67 ng/mL	0.01-3 ng/mL	Within normal range
Thyroid Stimulation hormone (TSH)	1.07 mIU/L	0.47-3.41 mIU/L	Within normal range
Insulin-like Growth Factor 1 (IGF-1)	165 ng/mL	100-300 ng/mL	Within normal range

**Figure 4:**
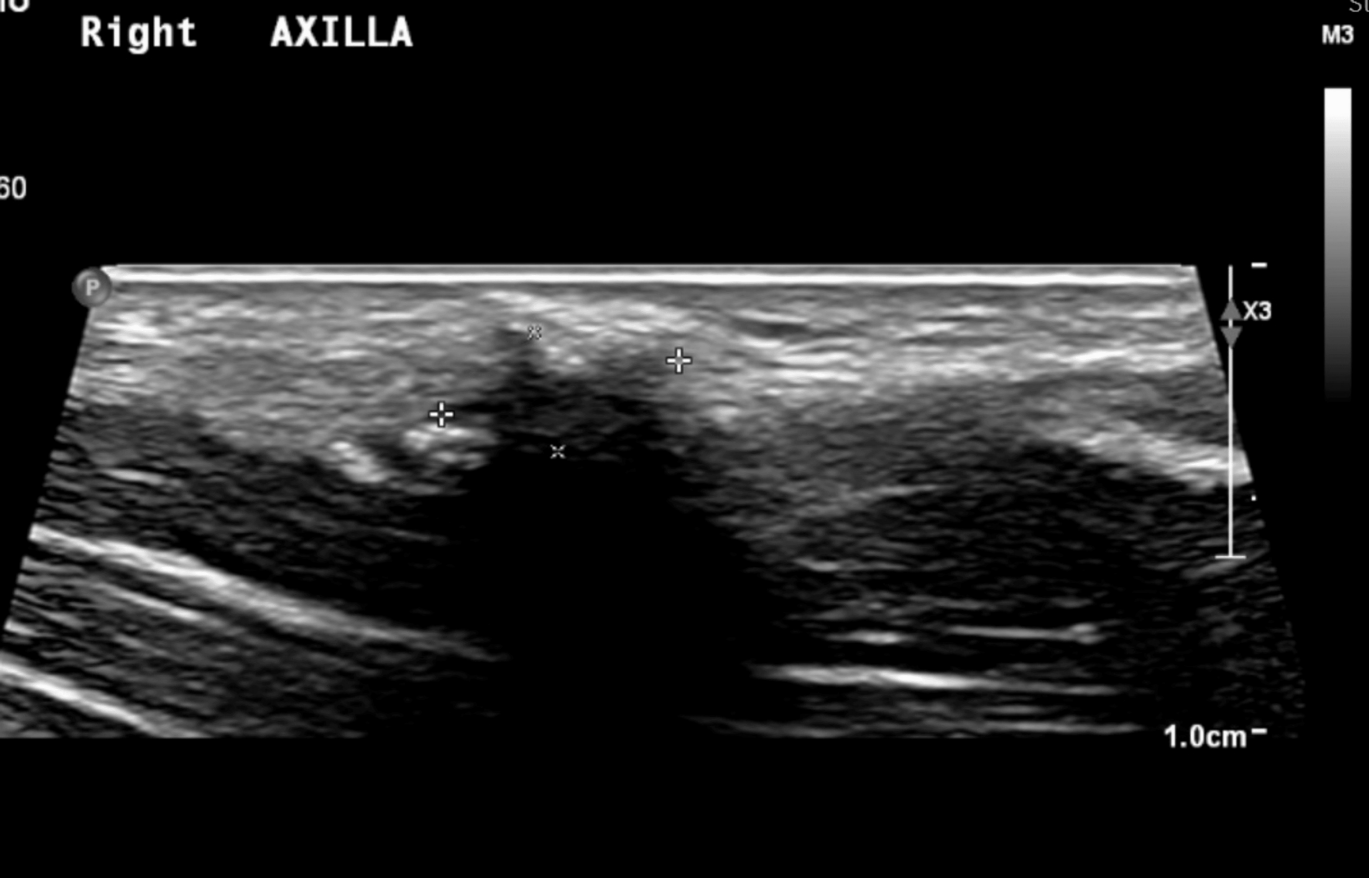
A multifocal subcutaneous lesion in the right axilla

**Figure 5:**
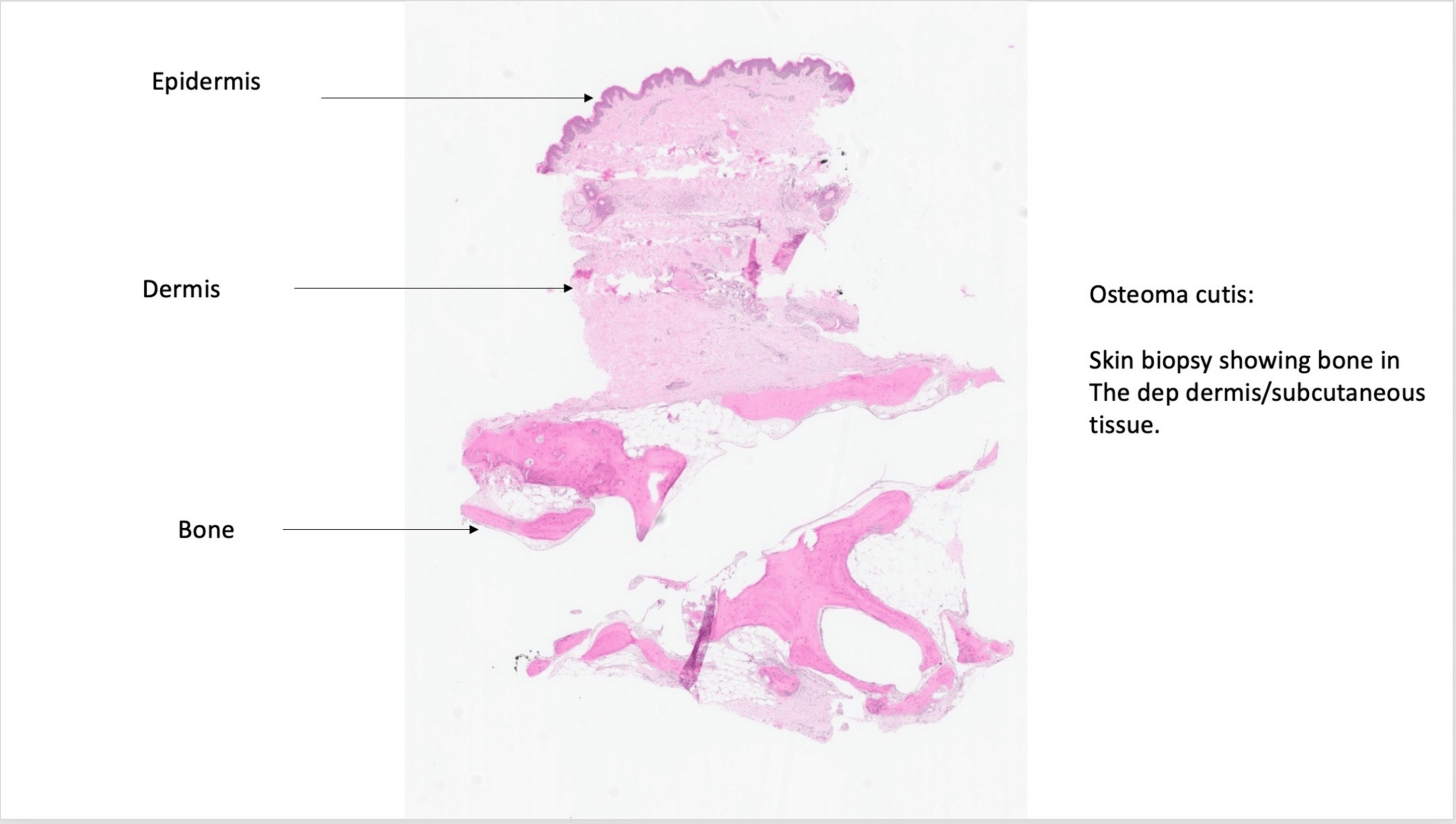
Skin biopsy showing bone in deep dermis/subcutaneous tissue

**Figure 6:**
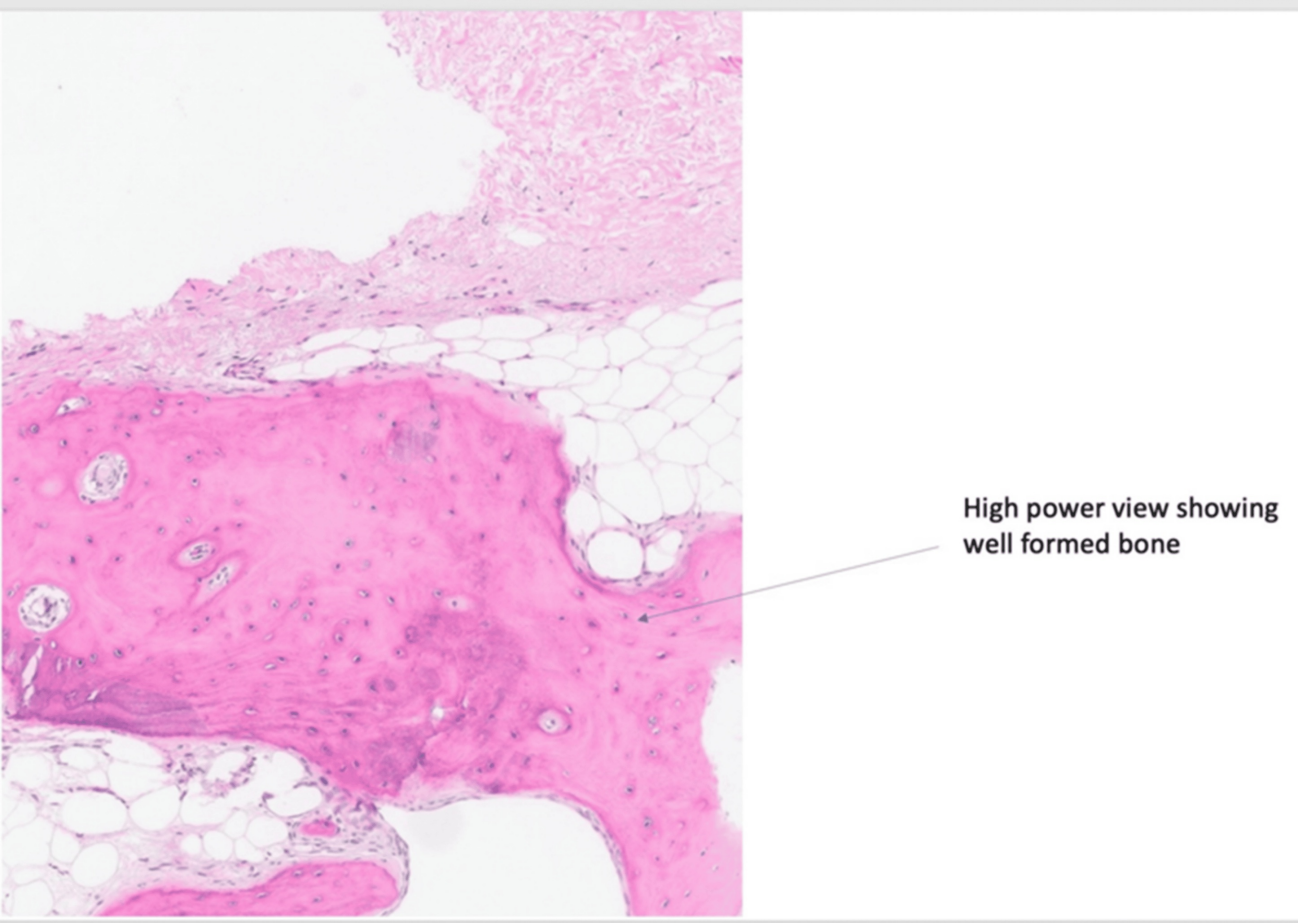
High power view showing well formed bone

An ultrasound (US) of the arm revealed a multifocal subcutaneous lesion (Figure 4). Fine-needle aspiration was conducted, and the pathology report indicated osteoma cutis (Figures 5, 6). No kidney stones were found on renal US.

Genetic testing indicated a possible diagnosis of autosomal dominant guanine nucleotide-binding protein, alpha stimulating activity polypeptide (GNAS)-related disorders with a heterozygous mutation in the GNAS gene (variant c.30009C>A p.(Tyr1003)). Whole-exome sequencing identified a heterozygous novel GNAS variant, c.3009C>A p.(Tyr1003*), resulting in a premature stop codon in exon 13 that is predicted to escape nonsense-mediated decay. The variant was classified as a variant of uncertain significance (VUS) with strong supporting evidence according to CENTOGENE’s (CENTOGENE GmbH, Rostock, Germany) implementation of American College of Medical Genetics and Genomics/Association for Molecular Pathology (ACMG/AMP) guidelines. The report emphasized that pathogenic GNAS variants are associated with a spectrum of autosomal dominant GNAS inactivation disorders, including PHP (types Ia, Ib, and Ic), PPHP, POH, and osteoma cutis, with phenotypic expression influenced by the parental origin of the affected allele. The methodology involved exome enrichment and high-throughput sequencing using the CentoXome MOx platform (CENTOGENE GmbH, Rostock, Germany) with extensive coverage and bioinformatic analysis aligned to the Genome Reference Consortium Human Build 37/Human Genome version 19 (GRCh37/hg19) reference genome. Variant interpretation incorporated phenotype correlation, family history, inheritance patterns, and American College of Medical Genetics and Genomics/Clinical Genome Resource (ACMG/ClinGen) classification criteria. No additional clinically relevant variants related to the patient’s phenotype or secondary findings were detected. The report recommended clinical correlation, parental targeted testing to determine the inheritance pattern and parental origin of the variant, periodic re-evaluation of sequencing data, and genetic counseling. Despite being categorized as a VUS, the identified novel GNAS mutation, together with the patient’s clinical presentation, biochemical abnormalities, and histopathological findings, strongly supports the diagnosis of an atypical GNAS-related PHP spectrum disorder and expands the currently reported mutational spectrum of GNAS-associated diseases.

Treatment

The patient was referred to plastic surgery for the surgical excision of his painful abdominal subcutaneous nodules. He was administered calcitriol 0.25 mcg and calcium carbonate 600 mg (each once daily) and advised to follow a low-phosphate diet.

Outcome and follow-up

Upon follow-up, the patient’s laboratory results were as follows (Table 2). Moreover, he underwent a surgical excision of the osteoma cutis. The surgery was uneventful, and following the procedure, the patient reported improvement in his pain and greater comfort during daily activities.

**Table 2:** Follow-up laboratory examination results SI: International System of Units.

Laboratory Test	Initial Value	Follow-up Value	Reference Range	Interpretation
Parathyroid Hormone (PTH)	104.90 pg/mL (SI: 11.1 pmol/L)	88.4 pg/mL (SI: 9.4 pmol/L)	16.04-60.38 pg/mL (SI: 1.7-6.4 pmol/L)	Improved but remained mildly elevated
Phosphorus	5.3 mg/dL (SI: 1.70 mmol/L)	4.9 mg/dL (SI: 1.57 mmol/L)	2.5-4.5 mg/dL (SI: 0.8-1.44 mmol/L)	Improved but remained mildly elevated
Adjusted Calcium (Adj Ca)	8.7 mg/dL (SI: 2.18 mmol/L)	9.1 mg/dL (SI: 2.27 mmol/L)	8.6-10.2 mg/dL (SI: 2.15-2.55 mmol/L)	Normalized on follow-up
Alkaline Phosphatase (ALP)	133 U/L	91 U/L	39-114 U/L	Normalized on follow-up

## Discussion

We report the case of a patient presenting with firm, skin-colored nodules that developed over a 10-year period. These progressive skin lesions were evaluated by the Dermatology team; no other systemic symptoms were present. A skin biopsy revealed findings consistent with osteoma cutis. Osteoma cutis is characterized by ossification within the dermis or subcutaneous tissue [3]. Thus, a metabolic evaluation for underlying bone and mineral disorders was performed, including assessment of serum calcium, phosphate, vitamin D, and PTH levels. Laboratory investigations revealed elevated PTH with hypocalcemia, findings suggestive of PTH resistance. This result prompted further evaluation for an underlying diagnosis of PHP. Multiple disorders are known to be associated with PHP; Table 3 summarizes the key clinical, biochemical, and genetic characteristics of these disorders [4-6].

**Table 3:** A summary of the key clinical, biochemical, and genetic characteristics of these disorders which associated with PHP AHO – Albright Hereditary Osteodystrophy; PHP – Pseudohypoparathyroidism; PPHP – Pseudopseudohypoparathyroidism; POH – Progressive Osseous Heteroplasia; GNAS – Guanine Nucleotide-Binding Protein, Alpha-Stimulating Subunit Gene; PTH – Parathyroid Hormone; TSH – Thyroid-Stimulating Hormone; GHRH – Growth Hormone-Releasing Hormone; Ca²⁺ – Calcium Ion; PO₄³⁻ – Phosphate Ion; cAMP – Cyclic Adenosine Monophosphate.

Disorder	Genetic Features	Biochemical Features	Hormone Resistance	AHO Features	Typical Clinical Presentation
PHP Type 1A (PHP1A)	Maternal GNAS coding mutations (exons 1–13)	Hypocalcemia, hyperphosphatemia, high PTH	Resistance to PTH, TSH, GHRH, gonadotropins and CNS neurotransmitters (leading to obesity and variable degrees of intellectual disability and developmental delay)	Present	Early-onset hypocalcemia with AHO phenotype
PHP Type 1C (PHP1C)	Similar to PHP1A but normal Gsα activity	Similar to PHP1A	Similar to PHP1A	Present	Similar to PHP1A phenotype and hormone resistance
PHP Type 1B (PHP1B)	Maternal GNAS methylation defects (DMRs)	Hypocalcemia, hyperphosphatemia, high PTH	Mainly PTH resistance, mild or no TSH resistance	Absent	PTH resistance without AHO features; renal PTH resistance
Pseudopseudohypoparathyroidism (PPHP)	Paternal GNAS coding mutations	Normal calcium and phosphate	No hormone resistance	Present (AHO features without hormone resistance)	AHO phenotype without biochemical abnormalities
Progressive osseous heteroplasia (POH)	Paternal GNAS mutations affecting XLαs or Gsα	Usually normal calcium/phosphate	Variable	Present with progressive deep ossification	Progressive ossification into deep tissues/muscle
Osteoma cutis (OC)	Similar to POH	Usually normal calcium/phosphate	Variable	. Extraskeletal ossification	Extraskeletal ossification limited to the dermis and subcutaneous tissues.

The patient did not present with obesity, short stature, brachydactyly, or mental deficiency, which are typically associated with AHO [4]. Thus, we investigated the underlying genetic mutation via whole-genome gene sequencing, which revealed a GNAS variant (c.3009C>A p.(Tyr1003)) potentially associated with the patient’s phenotype. This novel variant has not been previously reported in the literature. PHP1A is a rare genetic condition characterized by the physical features of AHO combined with reduced responsiveness to several hormones. These commonly include PTH and thyroid-stimulating hormone (TSH), and may also involve gonadotropins, calcitonin, and growth hormone-releasing hormone. The disorder results from inactivating variants in the maternally inherited GNAS gene, which encodes the stimulatory G protein alpha subunit (Gsα). In contrast, mutations affecting the paternal allele lead to PPHP, in which AHO features are present without associated hormone resistance [7]. Multiple pathogenic variants in GNAS have been reported in both PHP1A and PPHP. Approximately two-thirds are truncating variants, such as frameshifts, nonsense mutations, or splice-site changes, that either produce shortened proteins or result in transcript degradation via nonsense-mediated decay. These alterations impair Gsα function and underlie the clinical phenotype. Variants that cause protein elongation are rare, with only a few cases reported in the literature [7].

PHP1B is characterized clinically by renal resistance to PTH, resulting in hypocalcemia and hyperphosphatemia despite elevated circulating PTH levels. It has been suggested that affected individuals typically present with symptomatic hypocalcemia during the pubertal growth spurt, a period associated with increased calcium requirements [8,9].

Bone imaging in patients with PHP1B generally does not reveal features of chronic PTH elevation, such as osteitis fibrosa cystica, which is commonly seen in primary hyperparathyroidism [10]. However, some individuals with PHP1B exhibit skeletal abnormalities, including elevated serum alkaline phosphatase levels and subperiosteal bone resorption on radiographs [6]. Mild brachydactyly has also been described, although the complete manifestation of AHO features is typically absent, and intellectual development remains normal [5-10]. PTH resistance is the principal endocrine abnormality in PHP1B. Other end-organ hormone resistance, such as TSH resistance, is uncommon but has been reported in some individuals, typically presenting as mildly elevated TSH levels with normal thyroid hormone concentrations, consistent with subclinical TSH resistance [5].

PTH resistance is the defining abnormality in PHP. Our patient presented with hypocalcemia and low vitamin D and was treated with oral calcium supplementation and active vitamin D preparations (calcitriol or alfacalcidol), yielding improved laboratory findings; thus, the recommendations support continuing this management to maintain normal serum calcium and phosphate while allowing PTH levels to remain in the upper normal range to support renal calcium reabsorption and limit hypercalciuria. Excessively high or suppressed PTH levels should be avoided because of potential renal and skeletal complications [1]. Thus, management requires regular biochemical monitoring, prompt correction of symptomatic hypocalcemia, selective renal, neurological, and ophthalmological evaluation, and routine dental follow-up; treatment with PTH or PTH analogs is not recommended [1-11].

Impaired growth is common in PHP subtypes, except PHP1B, and is characterized by progressive deceleration of growth, advanced bone maturation, reduced pubertal growth, and early epiphyseal closure. As with many patients reported in the literature, Recombinant human GH is recommended for those with confirmed GH deficiency, while its use in other settings requires caution and individualized assessment, as long-term outcome data remain limited [12]. This case is noteworthy as the patient presented as underweight, with a BMI of 13, which contrasts with most PHP-related conditions; excess weight commonly occurs in individuals with PHP1A and PHP1B, often beginning in the first two years of life and sometimes representing the earliest or only clinical manifestation until later diagnosis [1]. Regular monitoring of BMI and eating behaviors is essential, along with early nutritional guidance, psychological support, and family education, even when weight is initially normal [12].

## Conclusions

Atypical presentations are sometimes seen in pseudohypoparathyroidism (PHP), even when the classical features of Albright hereditary osteodystrophy (AHO) are absent. Biochemical abnormalities suggestive of PTH resistance and the identification of a novel GNAS mutation supported the diagnosis within the PHP spectrum in our patient. This highlights the importance of considering genetic testing in patients with atypical presentations, as early identification of novel mutations may be helpful in guiding appropriate and timely management. PHP is usually associated with obesity, but some patients may show an underweight phenotype, which reflects the wide clinical spectrum of the condition. Some patients may benefit substantially from surgical procedures that improve symptoms and quality of life. In conclusion, PHP is complex and heterogeneous, and a multidisciplinary team approach is crucial to ensure accurate diagnosis and to provide holistic and patient-oriented treatment.
